# Supplementary material for: Comparative Efficacy and Safety of Immunotherapeutic Regimens with PD-1/PD-L1 Inhibitors for Previously Untreated Extensive-Stage Small Cell Lung Cancer: A Systematic Review and Network Meta-Analysis
Source: Curr Oncol. 2021 Feb 27;28(2):1094–113. doi: 10.3390/curroncol28020106 (PMC8025754; doi:10.3390/curroncol28020106)
Supplement: Supplementary file 1 [file curroncol-28-00106-s001.pdf]

Supplementary information

Comparative Efficacy and Safety of Immunotherapeutic Regimens with PD-1/PD-L1 Inhibitors for Previously Untreated Extensive-Stage Small Cell Lung Cancer: A Systematic Review and Network Meta-Analysis

Koichi Ando, Ryo Manabe, Yasunari Kishino, Sojiro Kusumoto, Toshimitsu Yamaoka, Akihiko Tanaka, Tohru Ohmori, Tsukasa Ohnishi and Hironori Sagara

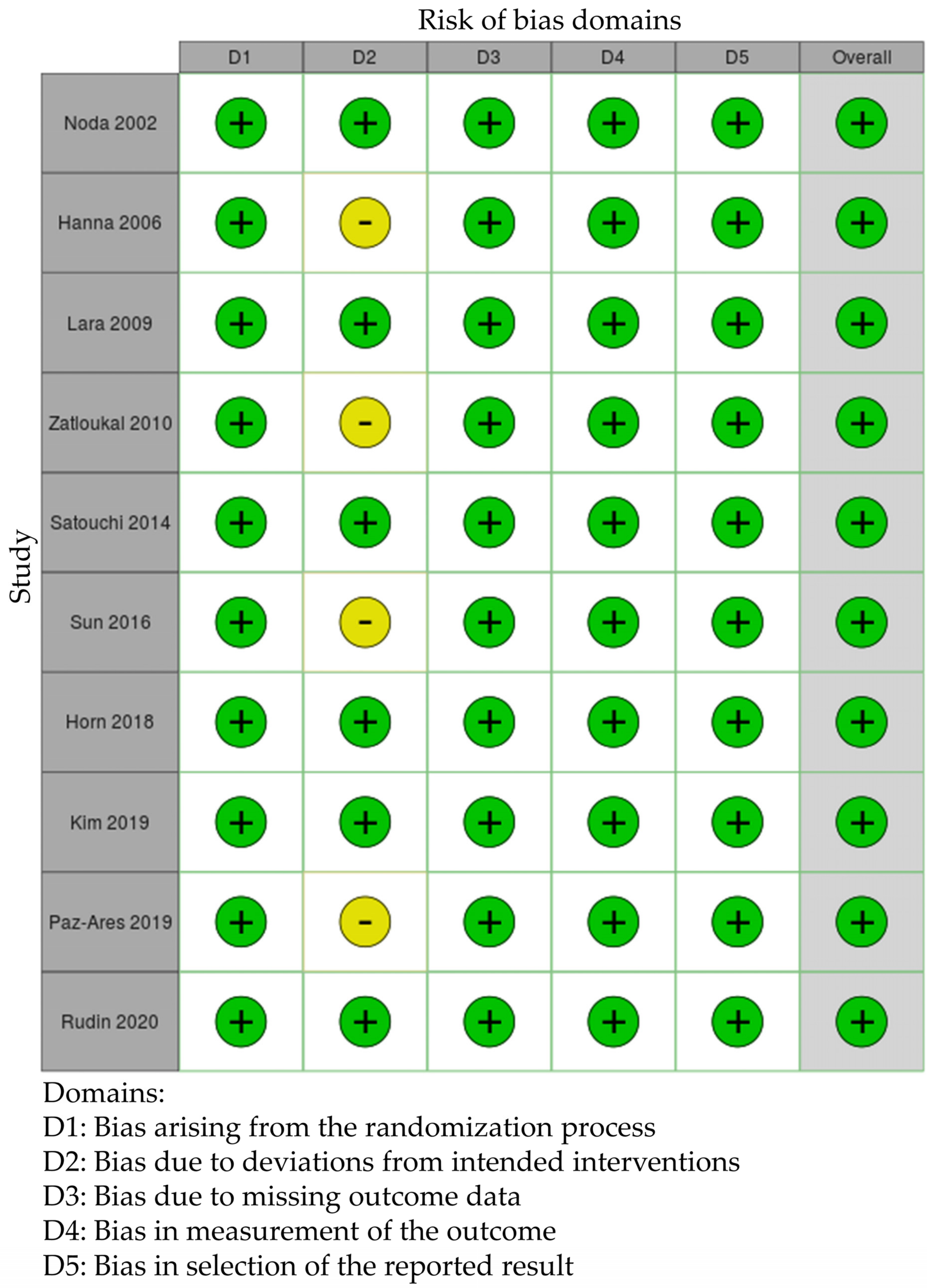

Figure S1. Risk of bias summary. The authors' judgments regarding each risk of bias item for each included study were reviewed. The symbols "+," "−," and "×" indicate a low risk of bias, some concerns, and a high risk of bias, respectively. The quality of the included studies was good because none of the studies were considered to have a high risk of bias, although four studies had some concerns regarding "bias due to deviations from intended interventions" as they were open-label studies.

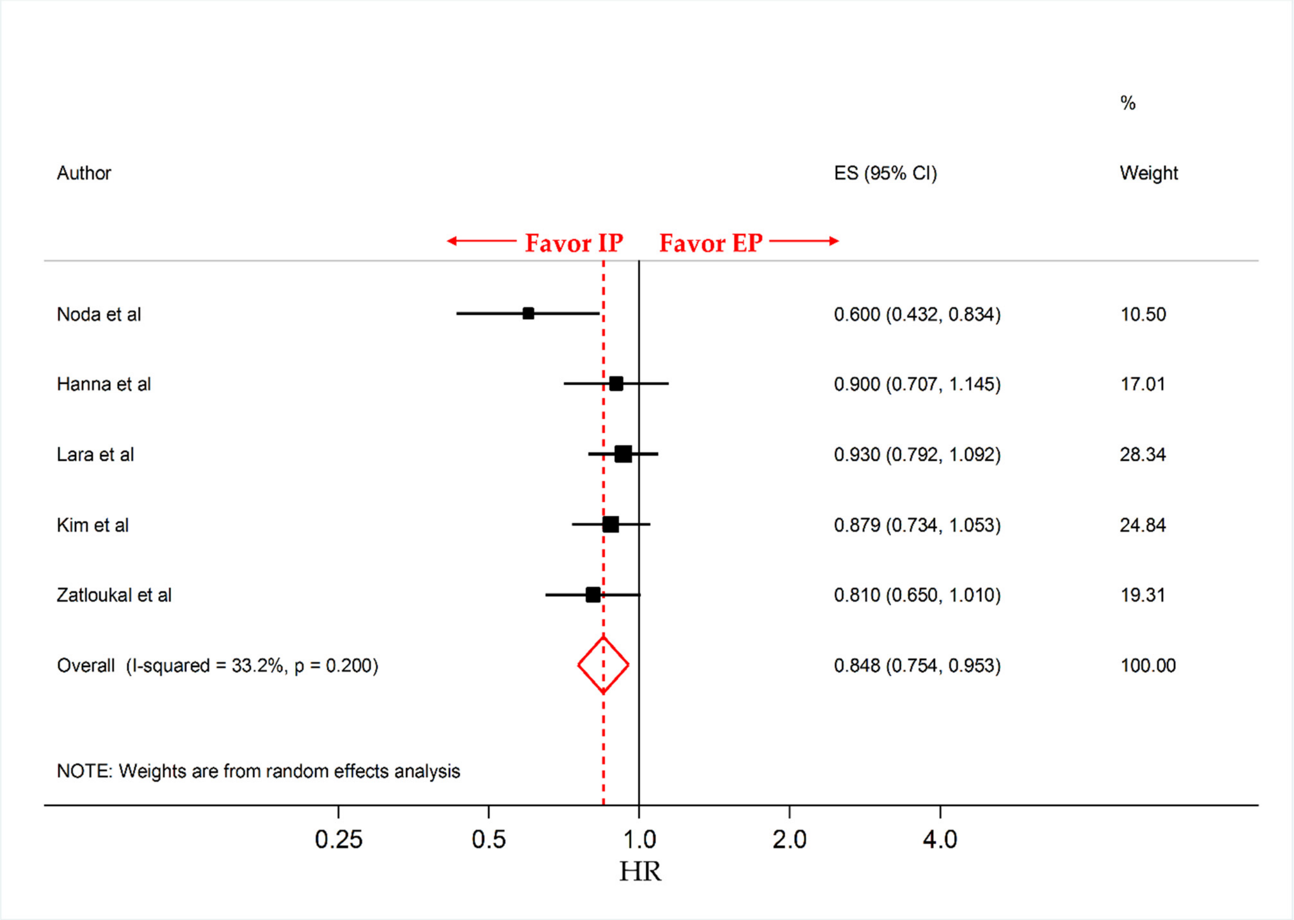

Figure S2. Forest plot for five trials comparing IP and EP. Meta-analysis of five trials comparing IP and EP for OS was performed based on random effect model, with assessment of heterogeneity as the main objective. Heterogeneity (I^2) was expressed as I-squared (%). Overall effect size (ES) for OS was expressed as HR and 95% CrI. The studies and data cited are from references 11, 15, 42-44. IP, platinum–irinotecan; EP, platinum–etoposide; HR, hazard ratio; CI, confidence interval; OS, overall survival.

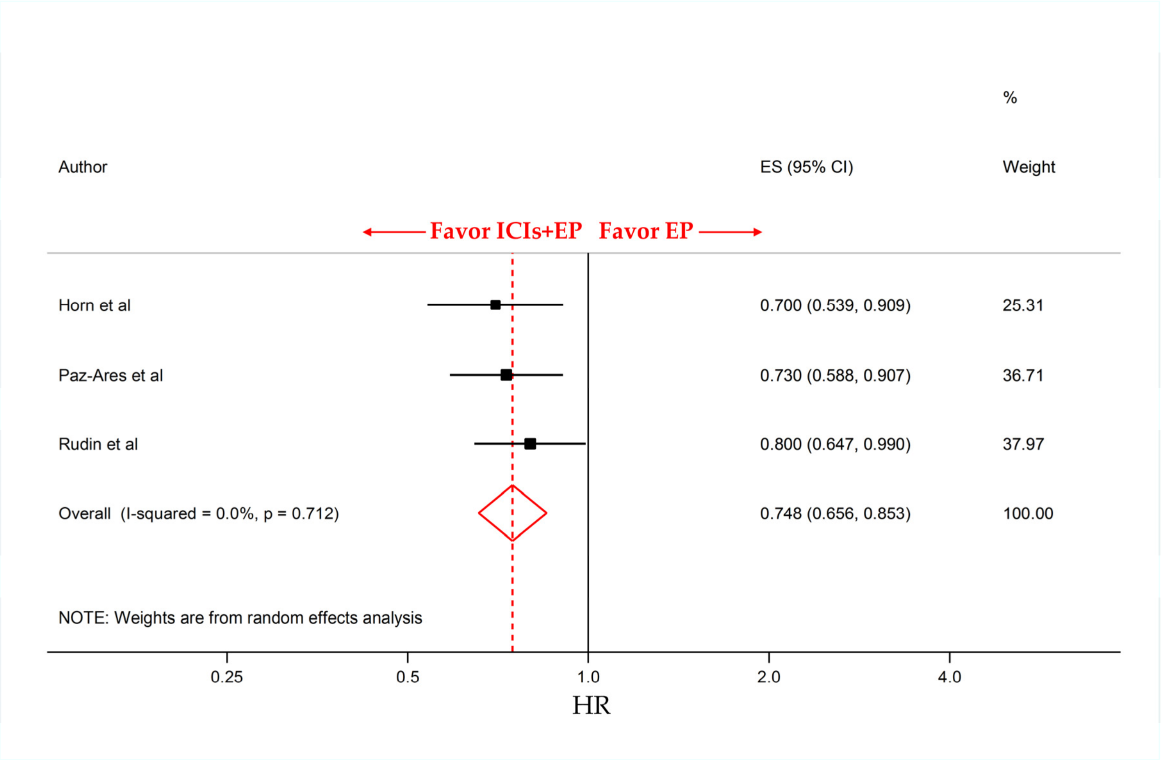

Figure S3. Forest plot for three trials comparing ICIs+EP and EP. Meta-analyses of the three trials comparing ICIs+EP and EP for OS were performed based on the random effect model, and assessment of heterogeneity was the main objective. Heterogeneity (I^2) is expressed as I-squared (%). The overall effect size (ES) for OS was expressed as HR and 95% CrI. The studies and data cited are from references 6–8. ICIs, immune checkpoint inhibitors; EP, platinum–etoposide; IP, platinum–irinotecan; HR, hazard ratio; CI, confidence interval; OS, overall survival.

Table S1. Key inclusion criteria of included studies

Author [Reference]	Study names	Year	Criteria
Noda et al. [15]	JCOG9511	2002	▪ ≤ 70 years of age<br>▪ ES-SCLC confirmed via histology or cytology<br>▪ No previous systemic anti-cancer treatment<br>▪ Performance status of 0 to 2
Hanna et al. [42]	–	2006	▪ ES-SCLC confirmed via histology or cytology<br>▪ No previous systemic anti-cancer treatment<br>▪ Performance status of 0 to 2
Lara et al. [11]	SWOG S0124	2009	▪ ES-SCLC confirmed via histology or cytology<br>▪ No previous systemic anti-cancer treatment<br>▪ Performance status of 0 or 1
Zatloukal et al. [43]	–	2010	▪ 18–75 years of age<br>▪ ES-SCLC confirmed using histology or cytology<br>▪ No previous systemic anti-cancer treatment<br>▪ Performance status of 0 or 1
Satouchi et al. [41]	JCOG0509	2014	▪ 20–70 years of age<br>▪ ES-SCLC confirmed using histology or cytology<br>▪ No previous systemic anti-cancer treatment<br>▪ Performance status of 0 or 1
Sun et al. [40]	NCT00660504	2016	▪ ≥ 18 years of age<br>▪ ES-SCLC confirmed using histology or cytology<br>▪ No previous systemic anti-cancer treatment<br>▪ Performance status of 0 or 1
Horn et al. [8]	IMpower133	2018	▪ ≥ 18 years of age<br>▪ ES-SCLC confirmed using histology or cytology<br>▪ No previous systemic anti-cancer treatment<br>▪ Performance status of 0 or 1
Kim et al. [44]	NCT00-349492	2019	▪ ≥ 18 years of age<br>▪ ES-SCLC confirmed using histology or cytology<br>▪ No previous systemic anti-cancer treatment<br>▪ Performance status of 0 to 2
Paz-Ares et al. [7]	CASPIAN	2019	▪ ≥ 18 years (20 years in Japan) of age<br>▪ ES-SCLC confirmed using histology or cytology<br>▪ No previous systemic anti-cancer treatment<br>▪ Performance status of 0 or 1
Rudin et al. [6]	KEYNOTE-604	2020	▪ ≥ 18 years of age

	· ES-SCLC confirmed using histology or cytology<br>· No previous systemic anti-cancer treatment<br>· Performance status of 0 or 1

Note: ES-SCLC, extensive-stage small-cell lung cancer.

Table S2. Characteristics of included studies

**Author year (Study name) [Reference]**	**P**	**Treatment arms (administration day within each cycle)**	**N**	**Age-yr median (range)**	**Female Sex N. (%)**	**ECOG PS N. (%)**	**PE**
Noda et al.<br>2002<br>(JCOG9511)<br>[15]	Ⅲ	Cis 60 mg/m^2 (1) plus<br>Iri 60 mg/m^2 (1, 8, 15), e4w	77	63 (30–70)	14 (18.2)	PS0: 10 (13.0)<br>PS1: 61(79.2)<br>PS2: 6 (7.8)	OS
		Cis 80 mg/m^2 (1) plus<br>Eto 100 mg/m^2 (1, 2, 3), e3w	77	63 (41–70)	8 (10.4)	PS0: 9 (11.7)<br>PS1: 58 (75.3)<br>PS2: 10 (13.0)	
		**total,**	**154**				
Hanna<br>2006<br>[42]	Ⅲ	Cis 30 mg/m^2 (1, 8) plus<br>Iri 65 mg/m^2 on day (1,8), e3w	221	63 (37–82)	NR (42.5)	PS0–1: NR (92.3)<br>PS2: NR (7.2)	OS
		Cis 60 mg/m^2 (1)<br>Eto 120 mg/m^2 (1, 2, 3), e3w	110	62 (38–83)	NR (42.7)	PS0–1: NR (88.2)<br>PS2: NR (10.9)	
		**total,**	**331**				
Lara<br>2009<br>(SWOG S0124)<br>[11]	Ⅲ	Cis 60 mg/m^2 (1) plus<br>Iri 60 mg/m^2 (1, 8, 15), e4w	324	62 (22–85)	136 (42)	NR	OS
		Cis 80 mg/m^2 (1) plus<br>Eto 100 mg/m^2 (1, 2, 3), e3w	327	63 (35–86)	145 (44)	NR	
		**total,**	**651**				
Zatloukal<br>2010<br>[43]	Ⅲ	Cis 80 mg/m^2 (1) plus<br>Iri 65 mg/m^2 (1, 8), e3w	202	60.0 (34–79)	48 (23.8)	PS0: 47 (23)<br>PS1: 153 (76)<br>PS2: 2 (1)	OS
		Cis 80 mg/m^2 (1) plus<br>Eto 100 mg/m^2 (1, 2, 3), e3w	203	61.0 (40–75)	48 (23.6)	PS0: 46 (23)<br>PS1: 157 (77)<br>PS2: 0 (0)	
		**total,**	**405**				
Satouchi<br>2014	Ⅲ	Cis 60 mg/m^2 (1) plus<br>Iri 60 mg/m^2 (1, 8, 15), e4w	142	63.0 (39–70)	22 (15.5)	PS0: 78 (54.9)<br>PS1: 64 (45.1)	OS

(JCOG0509)<br>[41]		Cis 60 mg/m² (1) plus<br>Amr 40 mg/m² (1, 2, 3), e3w	142	63.0 (29–70)	23 (16.2)	PS0: 80 (56.3)<br>PS1: 62 (43.7)	
		**total,**	**284**				
Sun<br>2016<br>(NCT00660504)<br>[40]	Ⅲ	Cis 60 mg/m² (1) plus<br>Amr 40 mg/m² (1, 2, 3), e3w	149	58.0 (13.0*)	35 (23.5)	PS0: 42 (28.2)<br>PS1: 107 (71.8)	OS
		Cis 80 mg/m² (1) plus<br>Eto 100 mg/m² (1, 2, 3), e3w	150	59.0 (13.0*)	37 (24.7)	PS0: 32 (21.3)<br>PS1: 118 (78.7)	
		**total,**	**299**				
Horn<br>2018<br>(IMpower133)<br>[8]	Ⅲ	Car AUC=5 (1) plus<br>Eto 100 mg/m² (1, 2, 3) plus<br>Atz 1200mg (1), e3w<br>[F/B Atz 1200mg (1), e3w]	201	64 (28–90)	72 (35.8)	PS0: 73 (36.3)<br>PS1: 128 (63.7)	OS<br>PFS
		Car AUC=5 (1) plus<br>Eto 100 mg/m² (1, 2, 3) plus<br>placebo (1), e3w<br>[F/B placebo (1), e3w]	202	64 (26–87)	70 (34.7)	PS0: 67 (33.2)<br>PS1: 135 (66.8)	
		**total,**	**403**				
Kim<br>2019<br>(NCT00-349492)<br>[44]	Ⅲ	Cis 70 mg/m² (1) plus<br>Iri 65 mg/m² (1, 8) e3w	173	66 (47–80)	22 (12.7)	PS0: 16 (9.2)<br>PS1: 132 (76.3)<br>PS2: 25 (14.4)	OS
		Cis 70 mg/m² (1) plus<br>Eto 100 mg/m² (1, 2, 3), e3w	189	65 (36–81)	12 (6.3)	PS0: 19 (10.0)<br>PS1: 141 (74.6)<br>PS2: 29 (15.3)	
		**total,**	**362**				
Paz-Ares<br>2019<br>(CASPIAN)<br>[7]	Ⅲ	[Car AUC=5–6 (1) or<br>Cis 75–80 mg/m² (1)] plus<br>Eto 80–100 mg/m² (1, 2, 3) plus<br>Dur 1,500mg (1), e3w<br>[F/B Dur 1,500 mg e4w)]	268	62 (58-68)	78 (29)	PS0: 99 (37)<br>PS1: 169 (63)	OS
		[Car AUC=5–6 (1) or	269	63 (57-68)	85 (32)	PS0: 90 (33)	

		Cis 75–80 mg/m^2 (1)] plus<br>Eto 80–100 mg/m^2 (1, 2, 3), e3w				PS1: 179 (67)	
		**total,**	**537**				
Rudin<br>2020<br>(KEYNOTE-604)<br>[6]	Ⅲ	[Car AUC=5 (1) or<br>Cis 75 mg/m^2 (1)] plus<br>Eto 100 mg/m^2 (1, 2, 3) plus<br>Pem 200 mg (1), e3w<br>[F/B Pem 200mg (1), e3w]	228	64 (24-81)	76 (33.3)	PS0: 60 (26.3)<br>PS1: 168 (73.7)	OS<br>PFS
		[Car AUC=5 (1) or<br>Cis 75 mg/m^2 (1)] plus<br>Eto 100 mg/m^2 (1, 2, 3) plus<br>placebo (1), e3w<br>[F/B placebo (1), e3w]	225	65 (37-83)	83 (36.9)	PS0: 56 (24.9)<br>PS1: 169 (75.1)	
		**total,**	**453**				

Note: P, phase; N, number of patients; yr, year; ECOG, Eastern Cooperative Oncology Group; PS, performance status; PE, primary endpoint; Cis, cisplatin, Iri, irinotecan; e4w, every 4 weeks; e3w, every 3 weeks; OS, overall survival; PFS, progression-free survival; Eto, etoposide; NR, not reported; Amr, Amrubicin; Car, carboplatin; AUC, area under the curve; Atz. Atezolizumab; F/B, followed by; Dur, durvalumab; Pem pembrolizumab: *, standard deviation.

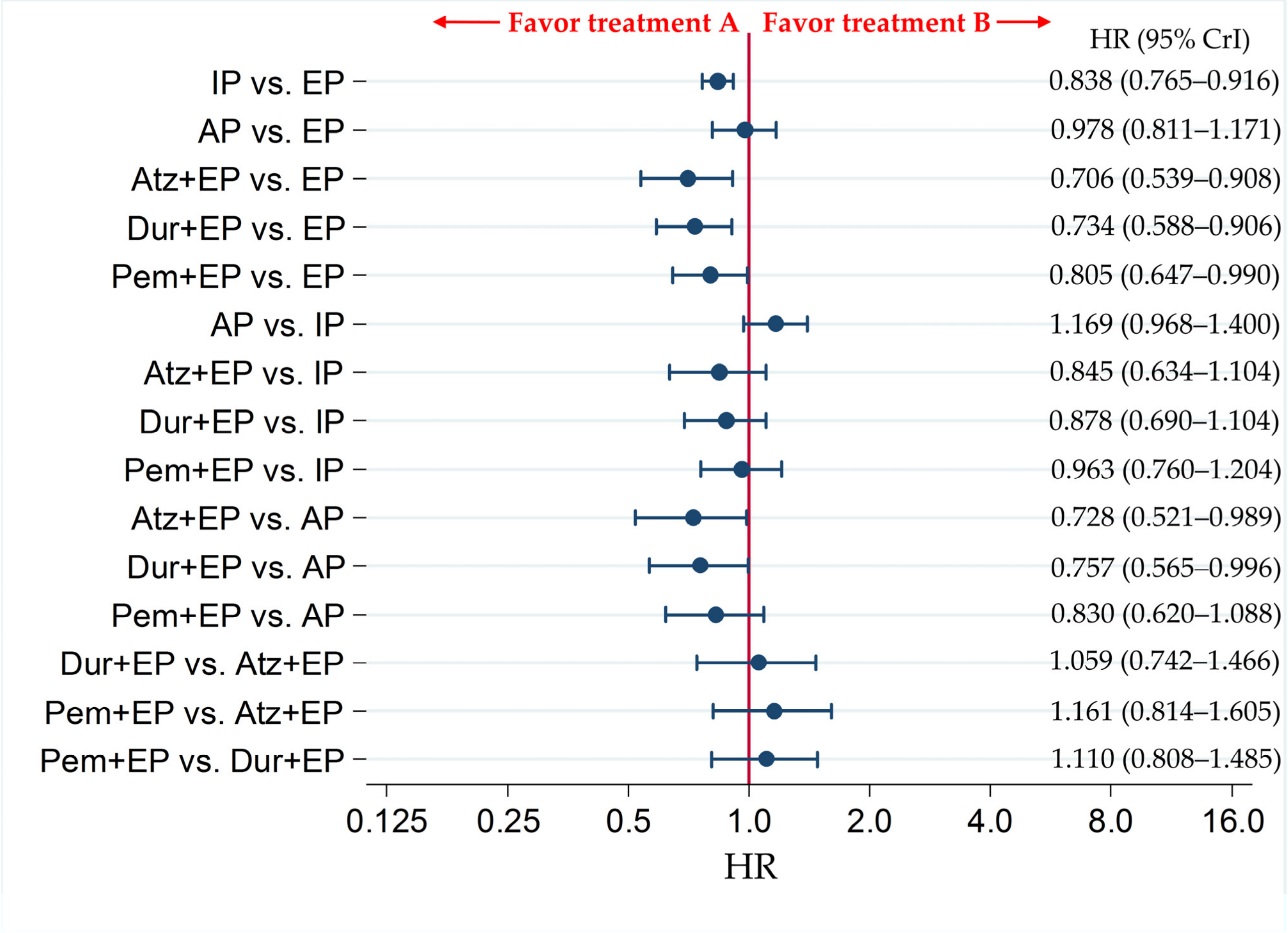

Figure S4. Comparative efficacy in terms of overall survival (OS) of each pair of treatments across six therapeutic regimens, including Pem+EP, Dur+EP, Atz+EP, AP, IP, and EP, for previously untreated ES-SCLC. Comparisons are shown as treatment A vs. treatment B. Data are expressed as hazard ratios and 95% credible intervals; Pem+EP, pembrolizumab plus platinum–etoposide; Dur+EP, durvalumab plus platinum–etoposide; Atz+EP; atezolizumab plus platinum–etoposide; AP, platinum–amrubicin; IP, platinum–irinotecan; EP, platinum–etoposide; HR, hazard ratio; CrI, credible interval; ES-SCLC, extensive-stage small-cell lung cancer.

Table S3. SUCRA and (rank) of each four treatment regimens, including ICIs+EP, AP, IP, and EP for the efficacy and safety outcomes

Treatments	OS	PFS	G3-AE	G3-NP	G3-AN	G3-TP	G3-DI
EP	13.0 (4)	28.6 (3)	17.6 (3)	33.2 (3)	43.4 (3)	49.7 (3)	84.5 (1)
IP	67.6 (2)	68.3 (2)	100.0 (1)	100.0 (1)	77.1 (1)	100.0 (1)	0.3 (4)
AP	22.5 (3)	4.8 (4)	NE	3.8 (4)	4.7 (4)	0.0 (4)	47.0 (3)
ICIs+EP	96.9 (1)	98.3 (1)	32.5 (2)	62.9 (2)	74.8 (2)	50.3 (2)	68.2 (2)

Note: Data presented are the surface under the cumulative ranking curve (SUCRA) for the efficacy in terms of overall survival (OS), progression-free survival (PFS), and safety in terms of ≥ grade 3 adverse events (G3-AEs), ≥ grade 3 neutropenia (G3-NP), ≥ grade 3 anemia (G3-AN), ≥ grade 3 thrombocytopenia (G3-TP), and ≥ grade 3 diarrhea (G3-DI) for the four therapeutic regimens (ICIs+EP, AP, IP, and EP) in patients with previously untreated ES-SCLC. Data are listed as SUCRA values with (rank). Higher SUCRA values indicate better outcomes; ICIs+EP, immune checkpoint inhibitors plus platinum–etoposide; AP, platinum–amrubicin; IP, platinum–irinotecan; EP, platinum–etoposide; NE, not evaluable; ES-SCLC, extensive-stage small-cell lung cancer.

Table S4. SUCRA and (rank) of each six treatment regimens, including Pem+EP, Dur+EP, Atz+EP, AP, IP, and EP for the efficacy and safety outcomes

Treatments	OS	PFS	G3-AE	G3-NP	G3-AN	G3-TP	G3-DI
EP	8.3 (6)	17.5 (5)	33.0 (3)	37.6 (4)	48.3 (3)	51.5 (3)	74.8 (1)
IP	50.6 (4)	48.5 (4)	96.7 (1)	95.0 (1)	68.7 (2)	94.9 (1)	5.1 (6)
AP	15.9 (5)	3.1 (6)	NE	12.3 (6)	13.4 (6)	1.1 (6)	46.3 (4)
Atz+EP	85.0 (1)	75.7 (2)	29.9 (4)	42.7 (3)	32.0 (5)	31.6 (5)	29.4 (5)
Dur+EP	79.5 (2)	73.8 (3)	70.9 (2)	81.9 (2)	97.7 (1)	82.2 (2)	71.5 (3)
Pem+EP	60.7 (3)	81.4 (1)	19.5 (5)	30.5 (5)	39.9 (4)	38.8 (4)	72.9 (2)

Note: Data presented are the surface under the cumulative ranking curve (SUCRA) for the efficacy in terms of overall survival (OS), progression-free survival (PFS), and safety in terms of ≥ grade 3 adverse events (G3-AEs), ≥ grade 3 neutropenia (G3-NP), ≥ grade 3 anemia (G3-AN), ≥ grade 3 thrombocytopenia (G3-TP), and ≥ grade 3 diarrhea (G3-DI) for the six therapeutic regimens (Pem+EP, Dur+EP, Atz+EP, AP, IP, and EP) in patients with previously untreated ES-SCLC. Data are listed as SUCRA values with (rank). Higher SUCRA values indicate better outcomes. Pem+EP, pembrolizumab plus platinum–etoposide, Dur+EP, durvalumab plus platinum–etoposide; Atz+EP; atezolizumab plus platinum–etoposide; AP, platinum–amrubicin; IP, platinum–irinotecan; EP, platinum–etoposide; NE, not evaluable; ES-SCLC, extensive-stage small-cell lung cancer.

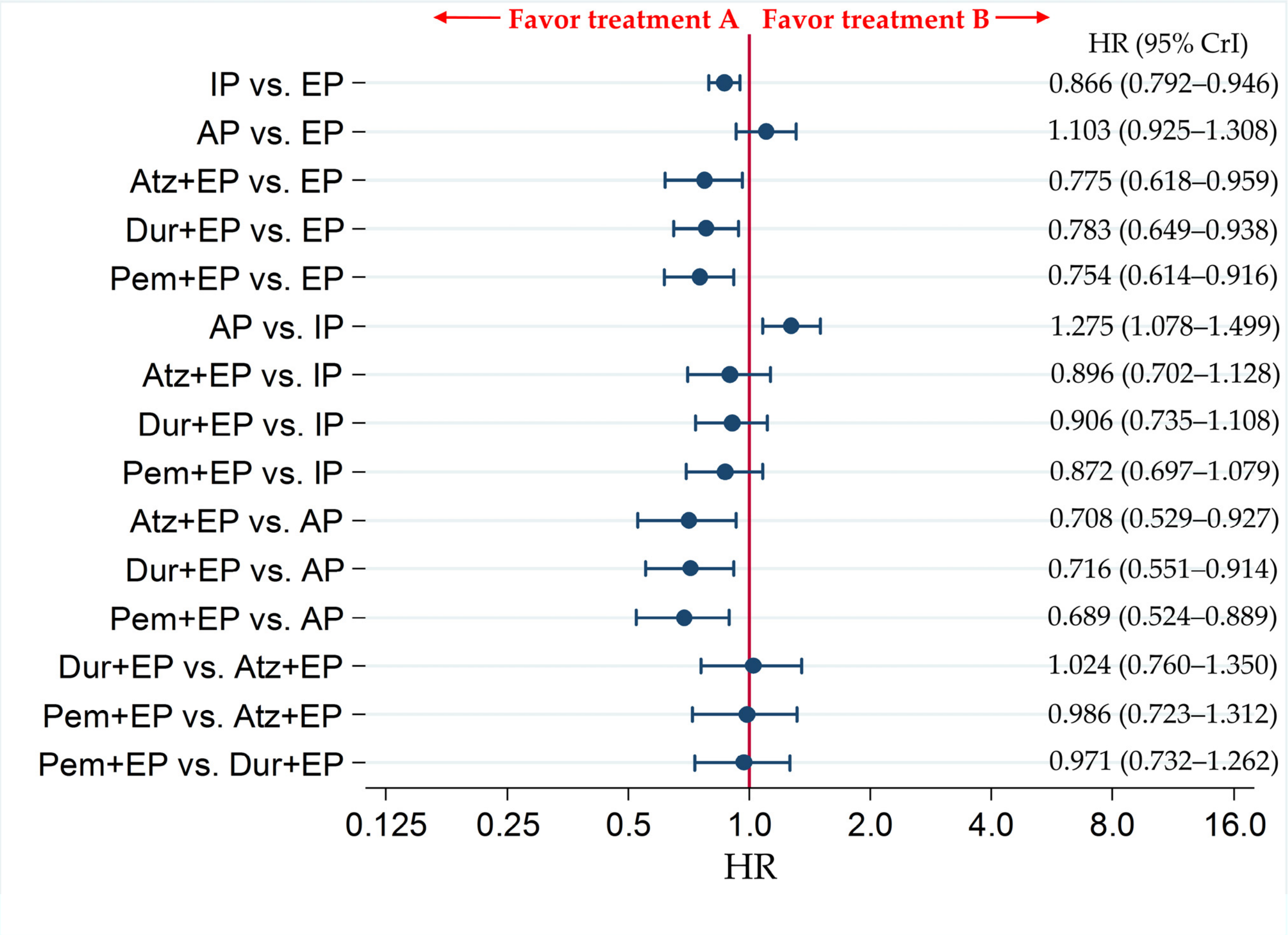

Figure S5. Comparative efficacy for progression free survival (PFS) of each treatment pair across six therapeutic regimens, including Pem+EP, Dur+EP, Atz+EP, AP, IP, and EP, for previously untreated ES-SCLC. Comparisons are shown as treatment A versus treatment B. The data are expressed as hazard ratios and 95% credible intervals. Pem+EP, pembrolizumab plus platinum–etoposide; Dur+EP, durvalumab plus platinum–etoposide; Atz+EP; atezolizumab plus platinum–etoposide; AP, platinum–amrubicin; IP, platinum–irinotecan; EP, platinum–etoposide; HR, hazard ratio; CrI, credible interval; ES-SCLC, extensive-stage small-cell lung cancer.

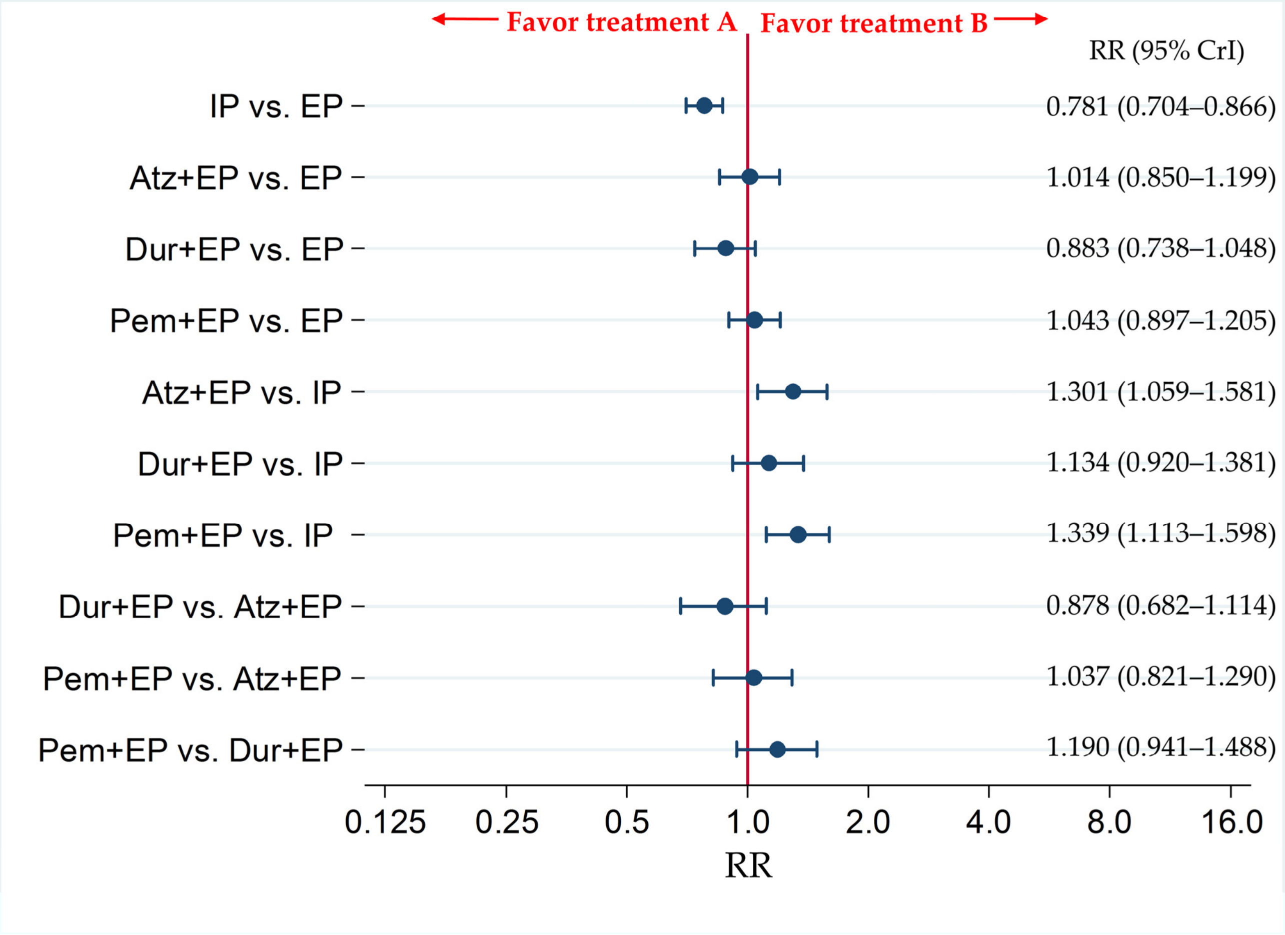

Figure S6. Comparative safety for ≥ grade 3 adverse events (G3-AEs) among the five therapeutic regimens, namely, Pem+EP, Dur+EP, Atz+EP, IP, and EP, for previously untreated ES-SCLC. Comparisons are shown as treatment A versus treatment B. Data are expressed as risk ratios and 95% credible intervals. Pem+EP, pembrolizumab plus platinum–etoposide; Dur+EP, durvalumab plus platinum–etoposide; Atz+EP; atezolizumab plus platinum–etoposide; IP, platinum–irinotecan; EP, platinum–etoposide; RR, risk ratio; CrI, credible interval; ES-SCLC, extensive-stage small-cell lung cancer.

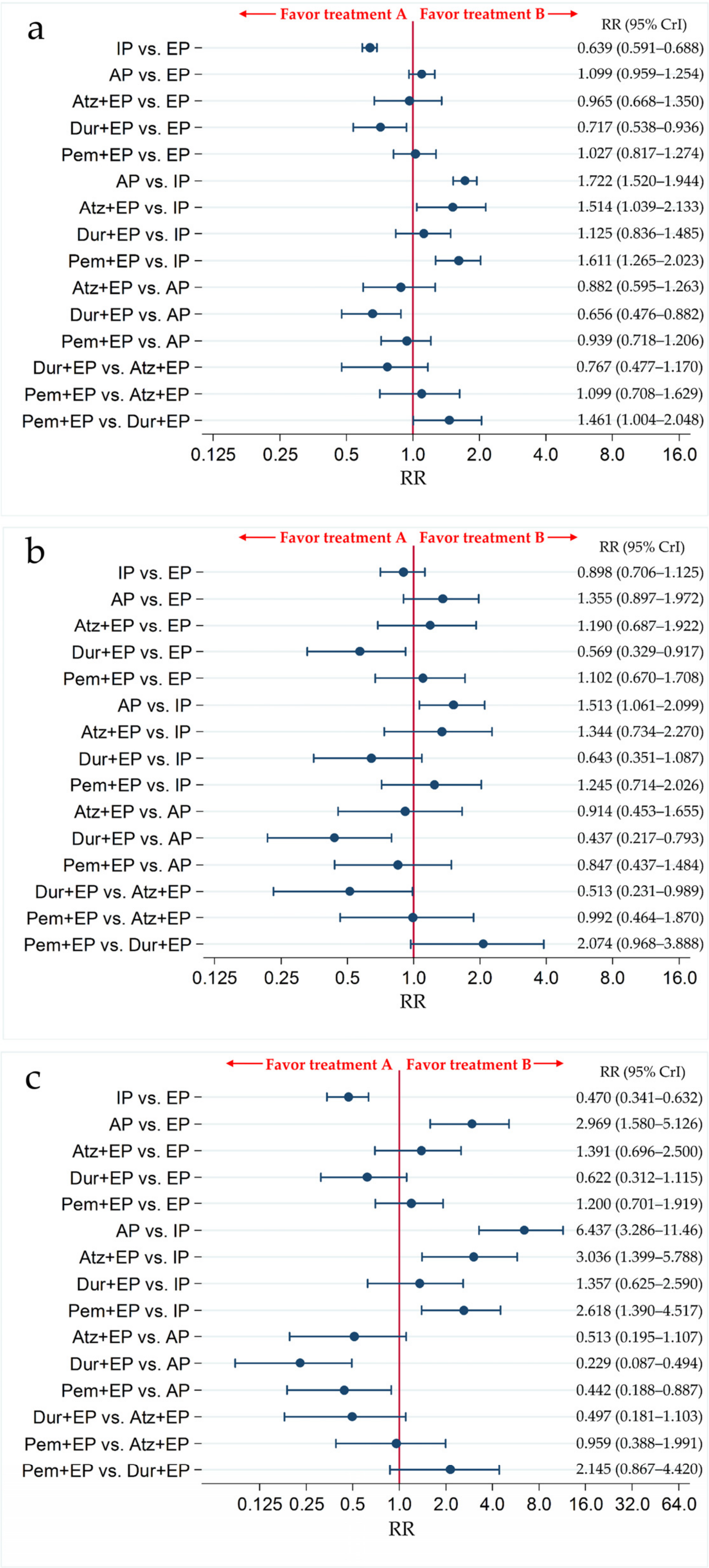

Figure S7. Comparative safety in terms of ≥ grade 3 **(a)** neutropenia, **(b)** anemia, and **(c)** thrombocytopenia among the six therapeutic regimens of Pem+EP, Dur+EP, Atz+EP, AP, IP, and EP for previously untreated ES-SCLC. Comparisons are

shown as treatment A versus treatment B. Data are expressed as risk ratios and 95% credible intervals. Pem+EP, pembrolizumab plus platinum–etoposide; Dur+EP, durvalumab plus platinum–etoposide; Atz+EP; atezolizumab plus platinum–etoposide; AP, platinum–amrubicin; IP, platinum–irinotecan; EP, platinum–etoposide; RR, risk ratio; CrI, credible interval; ES-SCLC, extensive-stage small-cell lung cancer.

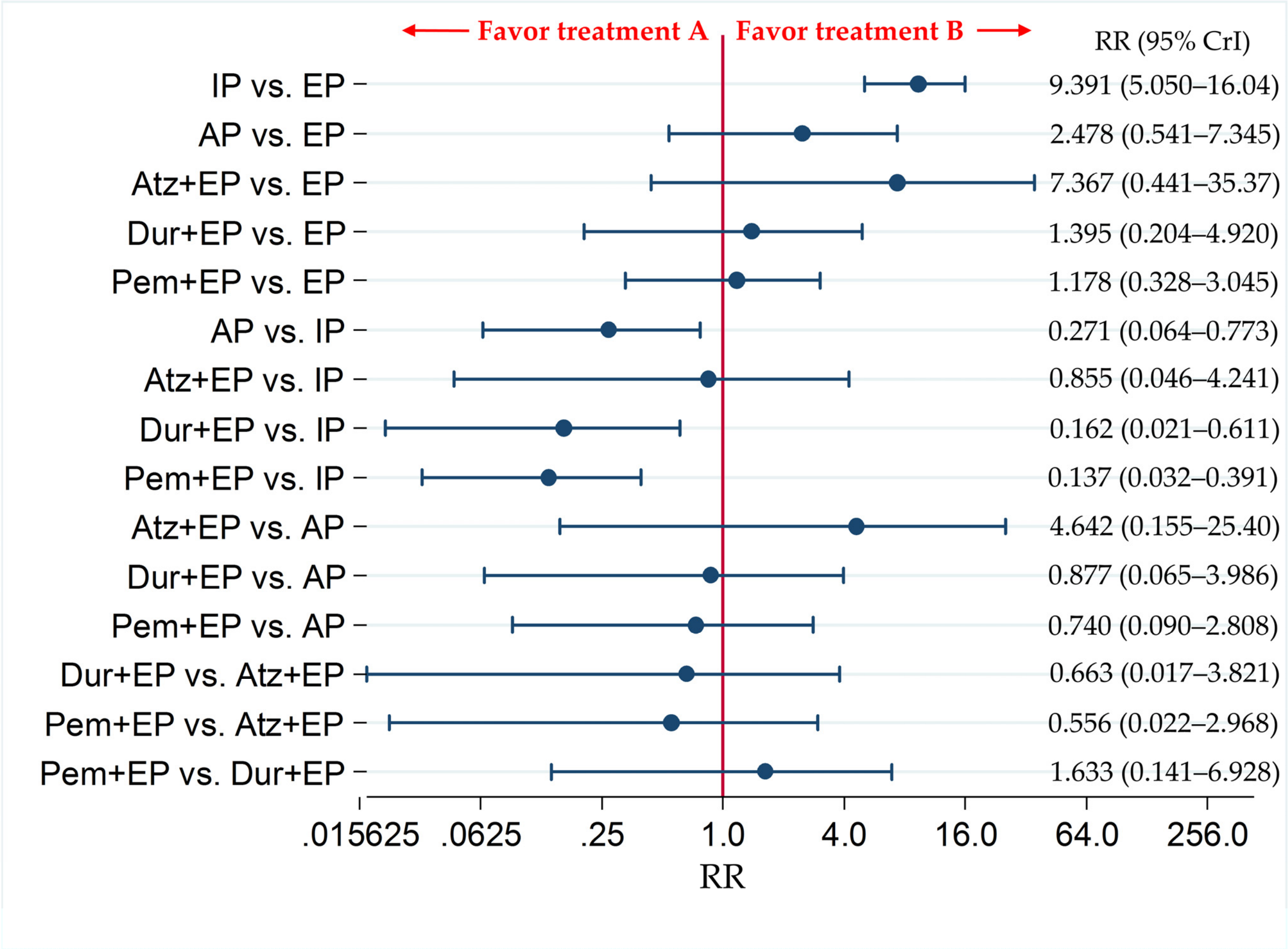

Figure S8. Comparative safety for G3-diarrhea of each treatment pair across six therapeutic regimens, including Pem+EP, Dur+EP, Atz+EP, AP, IP, and EP, for previously untreated ES-SCLC. Comparisons are shown as treatment A versus treatment B. The data are expressed as risk ratios and 95% credible intervals. Pem+EP, pembrolizumab plus platinum–etoposide; Dur+EP, durvalumab plus platinum–etoposide; Atz+EP; atezolizumab plus platinum–etoposide; AP, platinum–amrubicin; IP, platinum–irinotecan; EP, platinum–etoposide; RR, risk ratio; CrI, credible interval; ES-SCLC, extensive-stage small-cell lung cancer.

Table S5. Sensitivity analysis for OS based on the performance status (PS) among four treatment arms, including ICIs+EP, AP, EP, and IP

Treatment comparison	HR [95% CrI]
ICIs+EP vs. EP	0.749 [0.655–0.852]
ICIs+EP vs IP	0.892 [0.742–1.063]
ICIs+EP vs. AP	0.771 [0.609–0.962]

Note: To determine whether heterogeneity of the inclusion criteria based on the performance status (PS) among the studies

included in the present network meta-analysis affected the final conclusions, a sensitivity analysis for overall survival (OS) was conducted by excluding the three trials [15,42,44] that included patients with PS 0, 1, or 2 and by only including the remaining seven trials [6–8,11,40,41,43] that allowed the inclusion of only patients with a PS of 0 or 1. Comparisons were performed between ICIs+EP (combined population of Pem+EP, Dur+EP, and Atz+EP) and EP, between ICIs+EP and IP, and ICIs+EP and AP. Data are expressed as hazard ratio (HR) and 95% credible interval (CrI). ICIs+EP, immune checkpoint inhibitors plus platinum–etoposide; Pem+EP, pembrolizumab plus platinum–etoposide; Dur+EP, durvalumab plus platinum–etoposide; Atz+EP, atezolizumab plus platinum–etoposide; AP, platinum–amrubicin; IP, platinum–irinotecan; EP, platinum–etoposide.

Table S6. Sensitivity analysis for ranking assessment for OS based on the performance status (PS) among four treatment arms, including ICIs+EP, AP, EP, and IP

Treatment regimen	SUCRA (rank)
EP	13.5 (4)
IP	67.8 (2)
AP	22.4 (3)
ICIs+EP	96.4 (1)

Note: To determine whether heterogeneity of the inclusion criteria based on the performance status (PS) among the studies included in the present network meta-analysis affected the final conclusions, a sensitivity analysis for overall survival (OS) was conducted by excluding the three trials [15,42,44] that included patients with PS 0, 1, or 2 and by only including the remaining seven trials [6–8,11,40,41,43] that allowed the inclusion of only patients with PS of 0 or 1. The ranking assessment was performed among four treatment arms of ICIs+EP (combined population of Pem+EP, Dur+EP, and Atz+EP), EP, IP, and AP. Data are shown as surface under the cumulative ranking (SUCRA) values, which are listed with the rank presented in parentheses next to the corresponding SUCRA value. ICIs+EP, immune checkpoint inhibitors plus platinum–etoposide; Pem+EP, pembrolizumab plus platinum–etoposide; Dur+EP, durvalumab plus platinum–etoposide; Atz+EP, atezolizumab plus platinum–etoposide; AP, platinum–amrubicin; IP, platinum–irinotecan; EP, platinum–etoposide.

Table S7. Sensitivity analysis for OS based on the performance status (PS) among six treatment arms, including Pem+EP, Dur+EP, Atz+EP, AP, EP, and IP

Treatment comparison	HR [95% CrI]
IP vs. EP	0.843 [0.744–0.951]
AP vs. EP	0.981 [0.810–1.179]
Atz+EP vs. EP	0.706 [0.538–0.910]
Dur+EP vs. EP	0.734 [0.588–0.906]
Pem+EP vs EP	0.805 [0.647–0.990]
AP vs. IP	1.166 [0.961–1.404]
Atz+EP vs. IP	0.841 [0.624–1.112]

Dur+EP vs. IP	0.875 [0.677–1.115]
Pem+EP vs. IP	0.959 [0.745–1.216]
Atz+EP vs. AP	0.727 [0.519–0.990]
Dur+EP vs. AP	0.756 [0.562–0.997]
Pem+EP vs. AP	0.829 [0.617–1.089]
Dur+EP vs. Atz+EP	1.059 [0.742–1.466]
Pem+EP vs. Atz+EP	1.161 [0.814–1.605]
Pem+EP vs. Dur+EP	1.110 [0.808–1.485]

Note: To determine whether heterogeneity of the inclusion criteria based on the performance status (PS) among the studies included in the present network meta-analysis affected the final conclusions, a sensitivity analysis for overall survival (OS) was conducted by excluding the three trials [15,42,44] that included patients with PS 0, 1, or 2 and by only including the remaining seven trials [6–8,11,40,41,43] that allowed the inclusion of only patients with a PS of 0 or 1. Comparisons were performed between each pair of treatment arms of Pem+EP, Dur+EP, Atz+EP, AP, IP, and EP. Data are expressed as hazard ratio (HR) and 95% credible interval (CrI). Pem+EP, pembrolizumab plus platinum–etoposide; Dur+EP, durvalumab plus platinum–etoposide; Atz+EP, atezolizumab plus platinum–etoposide; AP, platinum–amrubicin; IP, platinum–irinotecan; EP, platinum–etoposide.

Table S8. Sensitivity analysis for ranking assessment for OS based on the performance status (PS) among six treatment arms, including Pem+EP, Dur+EP, Atz+EP, AP, EP, and IP

Treatment regimen	SUCRA (rank)
EP	8.6 (6)
IP	50.4 (4)
AP	15.8 (5)
Atz+EP	84.9 (1)
Dur+EP	79.4 (2)
Pem+EP	60.9 (3)

Note: To determine whether heterogeneity of the inclusion criteria based on the performance status (PS) among the studies included in the present network meta-analysis affected the final conclusions, a sensitivity analysis for overall survival (OS) was conducted by excluding the three trials [15,42,44] that included patients with PS 0, 1, or 2 and by only including the remaining seven trials [6–8,11,40,41,43] that allowed the inclusion of only patients with PS of 0 or 1. The ranking assessment was performed among six treatment arms of Pem+EP, Dur+EP, Atz+EP, EP, IP, and AP. Data are shown as surface under the cumulative ranking (SUCRA) values, which are listed with the rank presented in parentheses next to the corresponding SUCRA value. Pem+EP, pembrolizumab plus platinum–etoposide; Dur+EP, durvalumab plus platinum–etoposide; Atz+EP, atezolizumab plus platinum–etoposide; AP, platinum–amrubicin; IP, platinum–irinotecan; EP, platinum–etoposide.

Table S9. Sensitivity analysis for OS based on the geography among three treatment arms, including ICIs+EP, EP, and

IP

Treatment comparison	HR [95% CrI]
ICIs+EP vs EP	0.749 [0.655–0.853]
ICIs+EP vs. IP	0.844 [0.706–1.001]

Note: To determine whether heterogeneity of the inclusion criteria based on the geography among the studies included in the present network meta-analysis affected the final conclusions, a sensitivity analysis for overall survival (OS) was conducted by excluding the four trials [15,40,41,44] that had been performed in Asian countries, and by only including the remaining six trials [6–8,11,42,43] that had been international cooperative study or performed in Western countries. Because none of the six trials included AP arm in the treatment group, the AP arm was not included in this sensitivity analysis. Comparisons were performed between ICIs+EP (combined population of Pem+EP, Dur+EP, and Atz+EP) and EP, between ICIs+EP and IP. Data are expressed as hazard ratio (HR) and 95% credible interval (CrI). ICIs+EP, immune checkpoint inhibitors plus platinum–etoposide; Pem+EP, pembrolizumab plus platinum–etoposide; Dur+EP, durvalumab plus platinum–etoposide; Atz+EP, atezolizumab plus platinum–etoposide; IP, platinum–irinotecan; EP, platinum–etoposide.

Table S10. Sensitivity analysis for ranking assessment for OS based on the geography among three treatment arms, including ICIs+EP, EP, and IP

Treatment regimen	SUCRA (rank)
EP	1.1 (3)
IP	50.2 (2)
ICIs+EP	98.7 (1)

Note: To determine whether heterogeneity of the inclusion criteria based on the geography among the studies included in the present network meta-analysis affected the final conclusions, a sensitivity analysis for overall survival (OS) was conducted by excluding the four trials [15,40,41,44] that had been performed in Asian countries, and by only including the remaining six trials [6–8,11,42,43] that had been international cooperative study or performed in Western countries. Because none of the six trials included AP arm in the treatment group, the AP arm was not included in this sensitivity analysis. The ranking assessment was performed among three treatment arms of ICIs+EP (combined population of Pem+EP, Dur+EP, and Atz+EP), EP, and IP. Data are shown as surface under the cumulative ranking (SUCRA) values, which are listed with the rank presented in parentheses next to the corresponding SUCRA value. ICIs+EP, immune checkpoint inhibitors plus platinum–etoposide; Pem+EP, pembrolizumab plus platinum–etoposide; Dur+EP, durvalumab plus platinum–etoposide; Atz+EP, atezolizumab plus platinum–etoposide; IP, platinum–irinotecan; EP, platinum–etoposide.

Table S11. Sensitivity analysis for OS based on the geography among five treatment arms, including Pem+EP, Dur+EP, Atz+EP, EP, and IP

Treatment comparisons	HR [95%CrI]
IP vs. EP	0.891[0.793–0.999]
Atz+EP vs. EP	0.706[0.539–0.908]
Dur+EP vs. EP	0.734[0.587–0.906]
Pem+EP vs. EP	0.805[0.646–0.989]
Atz+EP vs. IP	0.795[0.592–1.045]
Dur+EP vs. IP	0.827[0.642–1.048]
Pem+EP vs. IP	0.906[0.706–1.147]
Dur+EP vs. Atz+EP	1.058[0.743–1.464]
Pem+EP vs. Atz+EP	1.160[0.817–1.597]
Pem+EP vs. Dur+EP	1.110[0.809–1.488]

Note: To determine whether heterogeneity of the inclusion criteria based on the geography among the studies included in the present network meta-analysis affected the final conclusions, a sensitivity analysis for overall survival (OS) was conducted by excluding the four trials [15,40,41,44] that had been performed in Asian countries, and by only including the remaining six trials [6–8,11,42,43] that had been international cooperative study or performed in Western countries. Because none of the six trials included AP arm in the treatment group, the AP arm was not included in this sensitivity analysis. The overall survival (OS) was performed compared between each pair of five treatment arms of Pem+EP, Dur+EP, Atz+EP, EP, and IP. Data are expressed as hazard ratio (HR) and 95% credible interval (CrI). Pem+EP, pembrolizumab plus platinum–etoposide; Dur+EP, durvalumab plus platinum–etoposide; Atz+EP, atezolizumab plus platinum–etoposide; IP, platinum–irinotecan; EP, platinum–etoposide.

Table S12. Sensitivity analysis for ranking assessment for OS based on the geography among five treatment arms, including Pem+EP, Dur+EP, Atz+EP, EP, and IP

Treatment regimen	SUCRA (rank)
EP	1.2 (5)
IP	31.9 (4)
Atz+EP	83.1 (1)
Dur+EP	76.7 (2)
Pem+EP	57.1 (3)

Note: To determine whether heterogeneity of the inclusion criteria based on the geography among the studies included in the present network meta-analysis affected the final conclusions, a sensitivity analysis for overall survival (OS) was conducted by excluding the four trials [15,40,41,44] that had been performed in Asian countries, and by only including the remaining six trials [6–8,11,42,43] that had been international cooperative study or performed in Western countries. Because none of the six trials included AP arm in the treatment group, the AP arm was not included in this sensitivity

analysis. The ranking assessment was performed among five treatment arms of Pem+EP, Dur+EP, Atz+EP, EP, and IP. Data are shown as surface under the cumulative ranking (SUCRA) values, which are listed with the rank presented in parentheses next to the corresponding SUCRA value. Pem+EP, pembrolizumab plus platinum–etoposide; Dur+EP, durvalumab plus platinum–etoposide; Atz+EP, atezolizumab plus platinum–etoposide; AP, platinum–amrubicin; IP, platinum–irinotecan; EP, platinum–etoposide.
